# Differential diagnosis model for tuberculous and malignant pleural effusion combining U-Net automatic segmentation and deep learning

**DOI:** 10.3389/fmed.2026.1855938

**Published:** 2026-09-01

**Authors:** Chang Song, Chun-Yan Zhao, Shu-Lin Song, Xue-Wen Huang, Hang-Biao Qiang, Xiao-Shi Lin, Zhen-Tao Huang, Zhou-Hua Xie, Qing-Dong Zhu

**Affiliations:** 1 Department of Tuberculosis, The Fourth People’s Hospital of Nanning, Nanning, Guangxi, China; 2 Clinical Medical School, Guangxi Medical University, Nanning, Guangxi, China; 3 Department of Radiology, The Fourth People’s Hospital of Nanning, Nanning, Guangxi, China

**Keywords:** deep learning, differential diagnosis, malignant pleural effusion, tuberculous pleural effusion, U-Net

## Abstract

**Background:**

We aimed to construct and validate an intelligent differential diagnosis model that integrates U-Net-based automatic segmentation with a deep learning classification model, and to evaluate its diagnostic performance and clinical value for distinguishing tuberculous pleural effusion (TPE) from malignant pleural effusion (MPE).

**Methods:**

A total of 281 patients with pleural effusion confirmed by etiological or pathological evidence between January 2018 and August 2025 were included, comprising 143 patients with TPE and 138 with MPE. First, a U-Net model was employed to automatically segment pleural lesion regions on chest computed tomography (CT) images and extract regions of interest (ROIs). Subsequently, based on the segmentation results, a radiomics model, a two-dimensional deep learning (DL2D) model, and a comprehensive model integrating clinical features were constructed. Multiple machine learning algorithms, including support vector machines (SVMs), random forests (RFs), and extremely randomized trees (ERTs), were utilized for model construction and comparison. Model performance was evaluated using the area under the receiver operating characteristic curve (AUC), sensitivity, specificity, calibration curves, decision curve analysis (DCA), and the integrated discrimination improvement (IDI) and net reclassification improvement (NRI) indices.

**Results:**

The U-Net segmentation model achieved Dice coefficients of 0.873 and 0.862 in the training and test sets, respectively, indicating good segmentation performance. In the test set, the comprehensive model demonstrated the best performance, with an AUC of 0.934 (95% CI 0.8733–0.9955), sensitivity of 0.875, and specificity of 0.900. Its performance was superior to that of the clinical model (AUC = 0.767), the radiomics model (AUC = 0.841), and the DL2D model (AUC = 0.776). DCA confirmed that the comprehensive model provided a higher net clinical benefit across a wide range of threshold probabilities. Furthermore, IDI and NRI analyses indicated that the comprehensive model significantly improved predictive performance relative to the individual models (*p* < 0.05).

**Conclusion:**

The model combining U-Net-based automatic segmentation with a deep learning classification model exhibited excellent and balanced diagnostic performance for differentiating TPE from MPE. It has the potential to provide an objective, stable, and scalable intelligent decision-support tool for clinical practice.

## 1 Introduction

Tuberculous pleural effusion (TPE) and malignant pleural effusion (MPE) are the two most common causes of pleural effusion (PE) in clinical practice. TPE results from pleural infection with *Mycobacterium tuberculosis* (MTB), while MPE most commonly arises from pleural metastases from malignancies such as lung cancer and malignant mesothelioma, or from primary pleural malignancies (1, 2). Their imaging findings often overlap substantially, as both can present with unilateral or bilateral pleural effusion, with or without pleural thickening. This overlap poses a significant challenge to differential diagnosis based solely on traditional imaging examinations. However, the treatment strategies and prognoses for these two conditions differ markedly. TPE requires standardized antituberculosis therapy, typically for 6 to 12 months, whereas management of MPE primarily focuses on local control, systemic antitumor therapy, and palliative care. Delayed diagnosis or misdiagnosis may therefore result in missed treatment opportunities and potentially irreversible clinical consequences. Currently, diagnostic approaches for tuberculosis include mycobacterial culture, acid-fast bacilli (AFB) testing, Xpert MTB/RIF, and T-SPOT. While culture has high specificity, it is time-consuming. AFB testing is rapid but has low sensitivity and limited discriminatory capability, and although Xpert MTB/RIF testing is recommended in clinical practice, its diagnostic sensitivity remains insufficient (3). Pleural effusion cytology and pleural biopsy are commonly regarded as reference standards for diagnosing MPE, but their sensitivity is limited, and some patients with MPE may have negative cytology results (4–6). Furthermore, invasive procedures are associated with risks such as pneumothorax, bleeding, and pain, and may be poorly tolerated by older patients or those in poor general health (7). Imaging examinations, particularly computed tomography (CT), are widely used in practice due to their non-invasive, convenient, and reproducible nature (8). Chest X-ray (CXR) remains the most commonly used initial imaging method for detecting pleural effusion and suspected tuberculosis, but its diagnostic specificity is limited and it cannot reliably distinguish between tuberculous and malignant causes. CT, because of its higher contrast resolution and multiplanar reconstruction capabilities, provides more detailed morphological information and is therefore widely used for further differential diagnosis. However, its diagnostic interpretation depends heavily on radiologists’ experience, resulting in considerable interobserver variability across different levels of expertise (9). Moreover, subtle texture differences and occult morphological features are often difficult to detect visually. There is therefore a clear need for an efficient, accurate, and reproducible method for differential diagnosis.

In recent years, rapid advances in artificial intelligence (AI) for medical image analysis have provided new opportunities to address this problem. As an important branch of deep learning, convolutional neural networks (CNNs) have shown strong performance in feature extraction and hierarchical representation learning, and in several thoracic imaging tasks, including pulmonary nodule detection, pneumonia differentiation, and tumor staging, their performance has approached or even exceeded that of human experts (10–13). Because of its encoder-decoder architecture and skip connections, U-Net has become one of the most widely used models for medical image segmentation. It enables precise and automatic delineation of lesion regions while reducing the workload and variability associated with manual annotation (14). Integrating U-Net-based automatic segmentation with deep learning (DL) classification models to construct an integrated “segmentation–diagnosis” workflow may therefore enable precise lesion localization and support more effective feature extraction and disease discrimination based on the segmented regions (15, 16).

Based on this background, the present study aimed to develop a differential diagnosis model that combines U-Net-based automatic segmentation with a two-dimensional deep learning (DL2D) classification model. U-Net was used to automatically and precisely segment pleural lesion regions on chest CT images to obtain regions of interest (ROIs). Based on these segmented regions, a DL model was constructed to extract deep, high-dimensional imaging features, thereby improving the accuracy of differentiation between TPE and MPE.

## 2 Materials and methods

### 2.1 Patient inclusion and diagnostic criteria

The study protocol was reviewed and approved by the Medical Ethics Committee of the Fourth People’s Hospital of Nanning (Ethics approval no. [2025] 52). The clinical and imaging data were strictly anonymized to ensure compliance with data usage regulations and the protection of patient privacy. Patients diagnosed with TPE and MPE between January 2018 and August 2025 were screened for eligibility. The diagnosis of TPE required meeting at least one of the following criteria (17, 18): (1) Positive MTB culture in PE or pleural biopsy specimens; (2) Positive nucleic acid detection for MTB (e.g., Xpert MTB/RIF or line probe assay) in PE or pleural biopsy specimens; (3) Pleural biopsy histopathology showing typical epithelioid granulomas with caseous necrosis and positive acid-fast staining. The diagnosis of MPE required meeting at least one of the following criteria: (1) pleural biopsy histopathology confirming malignancy, including primary malignant pleural mesothelioma, pleural metastasis from lung cancer, pleural metastasis from breast cancer, or pleural involvement by lymphoma; (2) malignant cells detected in pleural fluid cytology and confirmed by immunocytochemistry; or (3) pleural tissue obtained by thoracoscopy or thoracotomy with pathologic confirmation of malignant disease. The inclusion criteria were as follows: (1) complete clinical data, including detailed medical history, physical examination findings, and routine laboratory tests; (2) chest CT performed before treatment or within 72 h after treatment initiation; (3) definite PE on non-contrast chest CT, with the effusion occupying at least one-third of the intercostal space on one side or showing an effusion depth of at least 2 cm on ultrasonography, thereby ensuring sufficient fluid volume for subsequent segmentation and feature extraction; and (4) age ≥18 years, with an interval of no more than 7 days between clinical and imaging data acquisition. The exclusion criteria were as follows: (1) unclear etiology or coexistence of both TPE and MPE; (2) effusion caused by cardiac, hepatic, or renal insufficiency, autoimmune disease, or other non-target conditions such as chylothorax, empyema, fungal infection, or drug-induced pleuritis; (3) previous tuberculosis or receipt of systemic antituberculosis therapy or antitumor therapy, including chemotherapy, targeted therapy, or immunotherapy, within 1 month before enrollment; (4) invasive procedures such as thoracentesis, biopsy, or intrapleural drug instillation before CT acquisition, which could affect lesion identification because of secondary imaging changes; (5) prior pleurodesis or decortication resulting in irreversible pleural anatomic changes that could impair segmentation accuracy; (6) poor CT image quality, including motion, respiratory, or metal artifacts that precluded reliable lesion identification; or (7) incomplete key clinical data, including pathologic or etiologic confirmation, or incomplete follow-up records.

After application of these criteria, a total of 281 eligible patients were included, comprising 143 patients in the TPE group and 138 in the MPE group. The entire cohort was randomly divided into a training set and a test set at a ratio of 7:3. Detailed clinical information for the two groups is presented in Supplementary Table 1. All model development and hyperparameter tuning were performed in the training set, whereas final model performance was evaluated in the held-out test set.

### 2.2 Data collection

Clinical data were sourced from the hospital electronic medical record system and included demographic characteristics, laboratory test results, comorbidities, and clinical symptoms. Demographic characteristics included age, gender, height, weight, and body mass index (BMI). Laboratory test indicators were categorized into three groups: lymphocyte subset analysis (including absolute counts of CD3+, CD4+, and CD8 + T cells and the CD4/CD8 ratio); complete blood count parameters (including white blood cell count, red blood cell count, hemoglobin, platelet count, neutrophil count, lymphocyte count, monocyte count, eosinophil count, and basophil count); and biochemical and inflammatory markers (C-reactive protein, erythrocyte sedimentation rate, serum potassium, sodium, calcium, creatinine, glucose, triglycerides, aspartate aminotransferase, and albumin). Comorbidity data included hypertension, diabetes, chronic obstructive pulmonary disease, heart disease, hepatitis, anemia, coagulation dysfunction, cerebral infarction, and human immunodeficiency virus infection. Clinical symptoms mainly included fever, chest tightness, cough, expectoration, chest pain, dyspnea, and moist rales. All clinical data were independently verified by two researchers to ensure the accuracy and completeness of data extraction; in cases of disagreement, a third senior clinician reviewed and made the final adjudication.

Imaging data were obtained from the hospital’s Picture Archiving and Communication System (PACS). Image data from chest CT examinations performed within 1 month before diagnosis were collected for all patients. All CT scans were performed using multislice spiral CT equipment, with the scan range extending from the thoracic inlet to the level of the diaphragm. Acquisition parameters included a tube voltage of 120 kV. All imaging data were stored in the Digital Imaging and Communications in Medicine (DICOM) standard format. Two radiologists with intermediate or higher professional titles independently evaluated image quality, excluding cases in which the pleural region could not be clearly identified due to severe motion artifacts, respiratory artifacts, or artifacts from metal implants. Imaging data meeting quality requirements were exported in uncompressed DICOM format for subsequent U-Net segmentation and classification analysis.

### 2.3 Automated ROI segmentation

A segmentation-based approach was used to identify the region of interest (ROI) by generating annotation labels. A U-Net segmentation network was trained on these high-fidelity annotations to enable automatic ROI delineation. To ensure spatial consistency, all input images and associated labels were reoriented to the right-anterior-superior (RAS) anatomical axis. Image volumes were resampled to an isotropic resolution of 1 mm × 1 mm × 1 mm via bilinear interpolation, whereas segmentation labels were resampled using nearest-neighbor interpolation. Image intensities were linearly scaled to a range of −500 to 1,500 units, and selective cropping was applied to mitigate the impact of outliers.

A foreground mask derived from the original scan was used to exclude background pixels and restrict analysis to anatomically relevant regions. To balance positive and negative samples, subvolumes were selectively cropped. During training, real-time augmentation techniques, including random spatial transformations and cropping, were applied to increase data variability and improve robustness across different anatomical configurations. A hybrid Dice-cross-entropy loss (DiceCELoss) was used to address class imbalance:


$$\text{Loss}=-\frac{1}{N}\sum_{i=1}^{N}(\alpha \log(\frac{2}{\frac{1}{\in +\mathrm{Dic}e_{i}}+\frac{1}{\in +CE_{i}}})+(1-\alpha )\log(\in +\mathrm{Dic}e_{i}))$$

where N denotes the batch size, $\mathrm{Dic}e_{i}$ and $CE_{i}$ refer to the Dice and Cross-Entropy losses of the $i^{\mathrm{th}}$ sample, respectively, and α is a predefined weight. Unlabeled voxels were excluded by assigning zero weights, guiding the model to concentrate learning on annotated structures and enhancing segmentation precision.

Model optimization was performed using the Adam optimizer with an initial learning rate of 1 × 10^−3^. Training proceeded for a maximum of 600 epochs, with early stopping triggered if no improvement was observed across 32 successive validation epochs to reduce the risk of overfitting. During inference, a sliding-window strategy with a patch size of 96 × 96 × 64 voxels was utilized to scan the image volume for ROI localization. Post-processing involved the removal of isolated false-positive regions by retaining only the four largest connected components using MONAI’s Keep Largest Connected Component function. Model performance was assessed using the mean intersection over union (mIOU) and Dice Similarity Coefficient (DICE) (Supplementary Figure 1). The mIOU quantified the overlap between predicted and ground truth segmentations, offering a robust evaluation of regional alignment.


$$\text{Dice}(\mathrm{GT},\text{Pred})=\frac{2\times \mathrm{are}a_{\mathrm{GT}}\cap \mathrm{are}a_{\text{Pred}}}{\mathrm{are}a_{\mathrm{GT}}+\mathrm{are}a_{\text{Pred}}}$$

$$\text{mIOU}(\mathrm{GT},\text{Pred})=\frac{\mathrm{are}a_{\mathrm{GT}}\cap \mathrm{are}a_{\text{Pred}}}{\mathrm{are}a_{\mathrm{GT}}\cup \mathrm{are}a_{\text{Pred}}}$$

$$\mathrm{SA}=1-\frac{|R_{s}-T_{s}|}{R_{s}},\mathrm{OS}=\frac{O_{s}}{R_{s}+T_{s}},\mathrm{US}=\frac{U_{s}}{R_{s}+O_{s}}.$$

Employing both DICE and mIOU metrics allows for a comprehensive evaluation of our model’s segmentation accuracy, ensuring a robust assessment of its performance in delineating ROIs. The model with the best performance was applied to the entire dataset to achieve consistent and fully automated extraction of the lesion areas. Based on the provided table, the segmentation model achieved an overall Dice score of 0.873 on the training cohort and 0.862 on the test cohort, indicating good and consistent performance across both datasets (Supplementary Table 2).

### 2.4 Radiomics workflow

Radiomics features, including shape descriptors, first-order intensity statistics, and texture features derived from the gray-level co-occurrence matrix (GLCM), gray-level run-length matrix (GLRLM), gray-level size-zone matrix (GLSZM), and neighborhood gray-tone difference matrix (NGTDM), were extracted quantitatively from the segmented pleural lesion regions. Feature extraction followed the recommendations of the Image Biomarker Standardisation Initiative (IBSI) and was implemented using PyRadiomics (version 3.0.1). To construct a stable and informative feature set while minimizing overfitting, a multistep feature-selection strategy was applied. First, univariate analysis using the independent-samples t-test or Mann–Whitney U test, depending on data distribution, was used to identify features significantly associated with the outcome (*p* < 0.05). Second, pairwise Pearson correlation coefficients were calculated, and among highly correlated feature pairs (|r| > 0.9), the feature with weaker discriminative performance was removed. Third, the remaining features were ranked using the minimum redundancy–maximum relevance (mRMR) algorithm to maximize relevance to the outcome while minimizing redundancy. Finally, least absolute shrinkage and selection operator (LASSO) logistic regression was applied, with the regularization parameter tuned by 10-fold cross-validation. Features with non-zero coefficients were retained to construct the final radiomics signature. The radiomics signature was then modeled using several machine learning classifiers, including support vector machines (SVMs), random forests (RFs), and Extra Trees. Hyperparameters were optimized using grid search with 5-fold cross-validation. Model performance was evaluated using AUC, accuracy, sensitivity, and specificity.

### 2.5 2D deep learning framework

**Figure 1:**
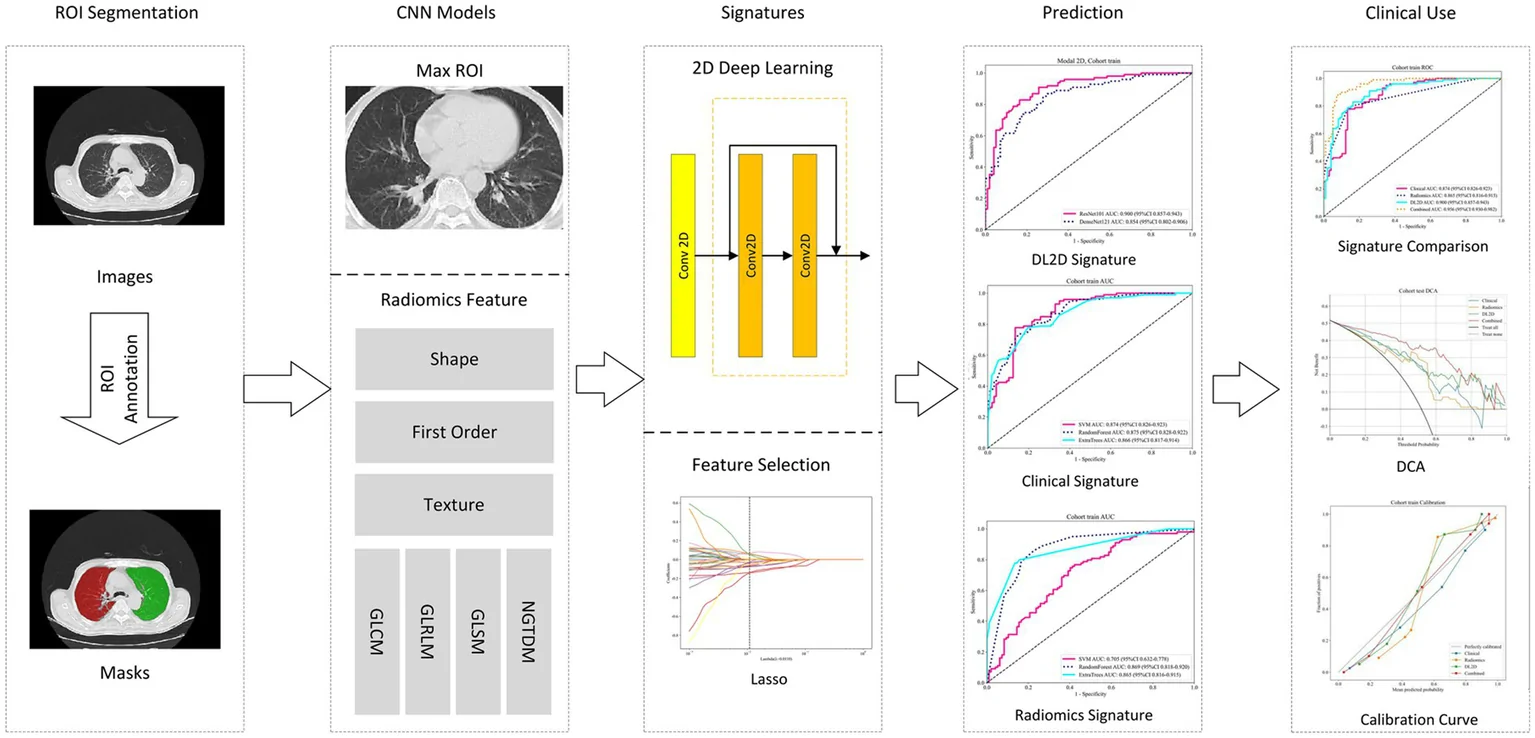
Research flowchart.

Input images were normalized using z-scores and scaled to a resolution of 224 × 224 pixels via nearest-neighbor interpolation. To enhance robustness, online data augmentation techniques, including random flipping and random cropping, were applied during training, whereas only normalization was used during inference. Model optimization was performed using stochastic gradient descent, and the loss function was softmax cross-entropy. The learning rate was dynamically adjusted using a cosine annealing schedule:


$$\eta_{t}=\eta_{\min}^{i}+\frac{1}{2}(\eta_{\max}^{i}-\eta_{\min}^{i})(1+\cos(\frac{T_{\mathrm{cur}}}{T_{i}}\pi ))$$

where $\eta_{\min}^{i}=0$ , $\eta_{\max}^{i}=0.01$ , and $T_{i}=32$ . Stochastic gradient descent (SGD) and softmax cross-entropy were used for optimization and loss computation, respectively. The probabilistic output generated by the best-performing deep learning model on the internal validation cohort was extracted and designated as the DL2D signature for each patient. Before integrating the three types of features (clinical features, radiomics features, and DL2D deep features), we performed independent standardization processing (z-score normalization) for each type of feature to eliminate the influence of differences in feature scales from different sources. The overall flowchart of this study is presented in Figure 1.

### 2.6 Statistical analysis

Normality was assessed using the Shapiro–Wilk test. Depending on data characteristics, continuous variables were compared using the independent-samples t test or Mann–Whitney U test, whereas categorical variables were analyzed using the chi-square test. Statistical analyses were performed using Python (version 3.7.12) and Statsmodels (version 0.13.2). Radiomics feature extraction was conducted using PyRadiomics (version 3.0.1), machine learning analyses were implemented using scikit-learn (version 1.0.2), and deep learning models were developed using PyTorch (version 1.11.0) with CUDA 11.3.1 and cuDNN 8.2.1 acceleration.

### 2.7 Quality control

To ensure data quality and the reliability of the results throughout the study, we implemented the following systematic quality control measures. All CT examinations were performed using multi-slice spiral CT scanners from the same manufacturer and model (GE Healthcare, USA). Scanning parameters strictly adhered to a standardized non-contrast chest protocol (tube voltage: 120 kV; tube current: automatic mA modulation), and equipment stability calibration was conducted monthly. All imaging data were exported in lossless DICOM format to ensure the integrity of the raw data. Among the 281 patients included in this study, all CT images met the diagnostic image quality standards, and no repeat scans were required due to equipment malfunction or abnormal acquisition parameters.

Two radiologists with intermediate professional titles or above (with 8 and 12 years of experience in chest imaging diagnosis, respectively) independently assessed the quality of each CT image. The evaluation criteria included: (a) the presence of severe motion or respiratory artifacts affecting the identification of the pleural or thoracic cavity regions; and (b) the presence of radial artifacts caused by metallic implants, specifically determining whether such artifacts obscured the pleural lesion areas. To ensure the reliability of radiological features, the intraclass correlation coefficient (ICC) was first calculated; only features with an ICC greater than 0.8, as assessed by the two physicians, were retained. This step ensured that only features demonstrating high consistency were subjected to further analysis. For correlation analysis, Pearson’s correlation coefficient was used to detect and remove highly correlated features, with a cutoff threshold of 0.9.

All clinical data were extracted from the electronic medical record system by two independent researchers and cross-verified. The extracted content included demographic characteristics, laboratory test results, comorbidities, and clinical manifestations. Data entry utilized a double-entry method by two personnel, followed by logical verification (e.g., checking age ranges and normal laboratory value ranges). Any discrepancies were reviewed and corrected by a third clinical expert (chief physician). The integrity and accuracy of the final dataset were confirmed through two independent audits.

Meanwhile, all models were optimized only on the training set, and the test set was used solely for the final performance evaluation. This ensured the objectivity and unbiased nature of the evaluation results.

## 3 Results

### 3.1 Construction of the clinical model

As shown in Table 1, the diagnosis of TPE was primarily established based on pleural biopsy revealing epithelioid granulomas with caseous necrosis (62.2%), positive MTB culture (46.9%), and positive Xpert MTB/RIF (36.4%). In contrast, the diagnosis of MPE was mainly confirmed by the detection of malignant cells in pleural effusion cytology (59.4%) and pleural biopsy demonstrating malignant tumor infiltration (55.1%). Supplementary Table 2 presents the results of the univariate and multivariate logistic regression analyses of the clinical variables, which identified nine independent predictors. Based on these predictors, a clinical model was constructed and evaluated using various machine learning algorithms. Table 2 compares the diagnostic performance of the clinical models in the training and test sets. In the training set, the AUCs of the SVM, RF, and ERT models were 0.874, 0.875, and 0.866, respectively, indicating good discriminative ability. In the test set, the corresponding AUCs were 0.822, 0.804, and 0.807, respectively. Overall, the SVM model achieved the highest AUC in the test set and showed the best overall discriminative performance and generalizability, with a sensitivity of 0.955.

**Table 1:** Comparison of diagnostic criteria between the TPE group and the MPE group.

Diagnostic modality	TPE (*n* = 143)	MPE (*n* = 138)
Microbiological test result		
MTB culture positive	67 (46.9%)	0 (0%)
Xpert MTB/RIF positive	52 (36.4%)	0 (0%)
Pleural biopsy histopathology		
Epithelioid granuloma with caseous necrosis	89 (62.2%)	0 (0%)
Infiltration of malignant tumor cells	0 (0%)	76 (55.1%)
Pleural fluid cytology		
Detection of malignant tumor cells	0 (0%)	82 (59.4%)
Other diagnostic methods	11 (7.7%)	21 (15.2%)
Those meeting multiple criteria (≥ 2 criteria)	76 (53.1%)	51 (37.0%)

TPE, tuberculous pleural effusion; MPE, malignant pleural effusion; MTB/RIF, *Mycobacterium tuberculosis*/rifampicin resistance.

**Table 2:** Clinical model performance of different machine learning algorithms in each cohort.

Model	AUC	95% CI	Sensitivity	Specificity	PPV	NPV	Cohort
SVM	0.874	0.826–0.923	0.778	0.866	0.856	0.792	train
SVM	0.822	0.735–0.909	0.955	0.537	0.689	0.917	test
RandomForest	0.875	0.828–0.922	0.737	0.845	0.830	0.759	train
RandomForest	0.804	0.712–0.896	0.727	0.756	0.762	0.721	test
Extra Trees	0.866	0.817–0.914	0.778	0.804	0.802	0.780	train
Extra Trees	0.807	0.718–0.897	0.818	0.634	0.706	0.765	test

AUC, area under the receiver operating characteristic curve; CI, confidence interval; PPV, positive predictive value; NPV, negative predictive value; SVM, support vector machine.

### 3.2 Construction of the radiomics model

A total of 1,834 radiomics features were extracted from the original images. Feature selection was performed using LASSO regression with 10-fold cross-validation to achieve sparse optimization of the feature space via penalized selection. Ultimately, eight features with non-zero coefficients were selected for model construction (Supplementary Figures 2A–C). The performance of the three machine learning algorithms was then compared in the training and test sets (Table 3). In the test set, the AUCs for the SVM, RF, and ERT models were 0.724, 0.726, and 0.828, respectively. Among these, the ERT model achieved the best overall performance, with a sensitivity of 0.841 and specificity of 0.756. The SVM model showed the highest specificity (0.878) but lower sensitivity, while the RF model showed a tendency toward overfitting.

**Table 3:** Radiomics model performance of different machine learning algorithms in each cohort.

Model	AUC	95% CI	Sensitivity	Specificity	PPV	NPV	Cohort
SVM	0.705	0.632–0.778	0.747	0.598	0.655	0.699	train
SVM	0.724	0.617–0.831	0.477	0.878	0.808	0.61	test
RandomForest	0.869	0.818–0.920	0.859	0.773	0.794	0.843	train
RandomForest	0.726	0.622–0.829	0.727	0.683	0.711	0.7	test
Extra Trees	0.865	0.816–0.915	0.778	0.866	0.856	0.792	train
Extra Trees	0.828	0.745–0.911	0.841	0.756	0.787	0.816	test

AUC, area under the receiver operating characteristic curve; CI, confidence interval; PPV, positive predictive value; NPV, negative predictive value; SVM, support vector machine.

### 3.3 Results of the DL2D model and signature

Table 4 illustrates the diagnostic performance of the DL2D model in the training and test sets. For the ResNet10, the AUCs in the training and test sets were 0.900 and 0.884, respectively. For the DenseNet121 model, they were 0.854 and 0.858, respectively. In the test set, ResNet101 achieved a specificity of 0.927, significantly superior to the 0.829 of DenseNet121, whereas DenseNet121 exhibited higher sensitivity in the test set (0.795 vs. 0.705).

**Table 4:** Performance metrics of the two-dimensional deep learning (DL2D) models.

Model	AUC	95% CI	Sensitivity	Specificity	PPV	NPV	Cohort
ResNet101	0.900	0.8567–0.9432	0.828	0.835	0.837	0.827	train
ResNet101	0.884	0.8134–0.9537	0.705	0.927	0.912	0.745	test
DenseNet121	0.854	0.8019–0.9062	0.848	0.722	0.757	0.824	train
DenseNet121	0.858	0.7769–0.9382	0.795	0.829	0.833	0.791	test

DL2D, two-dimensional deep learning; AUC, area under the receiver operating characteristic curve; CI, confidence interval; PPV, positive predictive value; NPV, negative predictive value.

### 3.4 Comparison of signatures

Table 5 compares the predictive performance of the clinical model, radiomics model, DL2D model, and the multimodal combined model in the training and test sets. In the training set, the combined model showed the best performance, with an AUC of 0.938 (95% CI 0.8991–0.9769), significantly outperforming the individual models. Its sensitivity and specificity were 0.915 and 0.946, respectively. By comparison, the AUCs of the clinical model, radiomics model, and DL2D model were 0.790, 0.856, and 0.830, respectively. In the test set, the combined model maintained the best performance, achieving an AUC of 0.934 (95% CI 0.8733–0.9955), with sensitivity and specificity of 0.875 and 0.900, respectively, demonstrating excellent generalizability and stability. Although the radiomics model and DL2D model also demonstrated high diagnostic value in the test set, with AUCs of 0.841 and 0.776, respectively, the combined model achieved maximized diagnostic efficacy by integrating multi-source information, validating the superiority of this comprehensive strategy in clinical applications.

**Table 5:** Comparison of predictive performance among clinical, radiomics, DL2D, and combined signatures in the training and test cohorts.

Signature	AUC	95% CI	Sensitivity	Specificity	PPV	NPV	Cohort
Clinical	0.790	0.7222–0.8577	0.596	0.978	0.966	0.705	train
Radiomics	0.856	0.8013–0.9099	0.936	0.667	0.739	0.912	train
DL2D	0.830	0.7723–0.8873	0.777	0.774	0.777	0.774	train
Combined	0.938	0.8991–0.9769	0.915	0.946	0.945	0.917	train
Clinical	0.767	0.6554–0.8783	0.675	0.900	0.871	0.735	test
Radiomics	0.841	0.7557–0.9262	0.925	0.650	0.725	0.897	test
DL2D	0.776	0.6758–0.8754	0.900	0.525	0.655	0.840	test
Combined	0.934	0.8733–0.9955	0.875	0.900	0.897	0.878	test

DL2D, two-dimensional deep learning; AUC, area under the receiver operating characteristic curve; CI, confidence interval; PPV, positive predictive value; NPV, negative predictive value.

### 3.5 Model performance evaluation and clinical application

**Figure 2:**
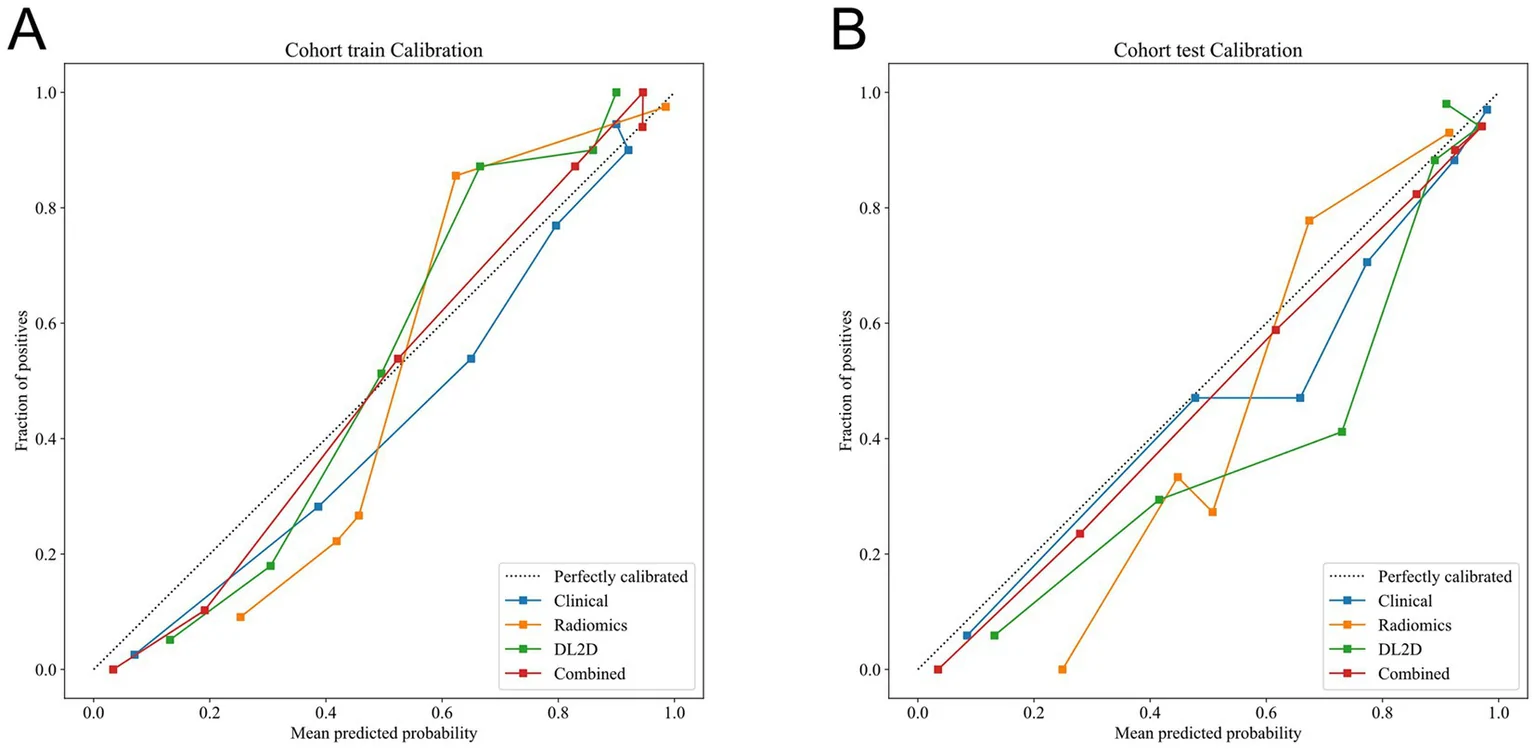
Model calibration comparison. **(A)** Calibration curve of the training cohort; **(B)** Calibration curve of the testing cohort.

**Figure 3:**
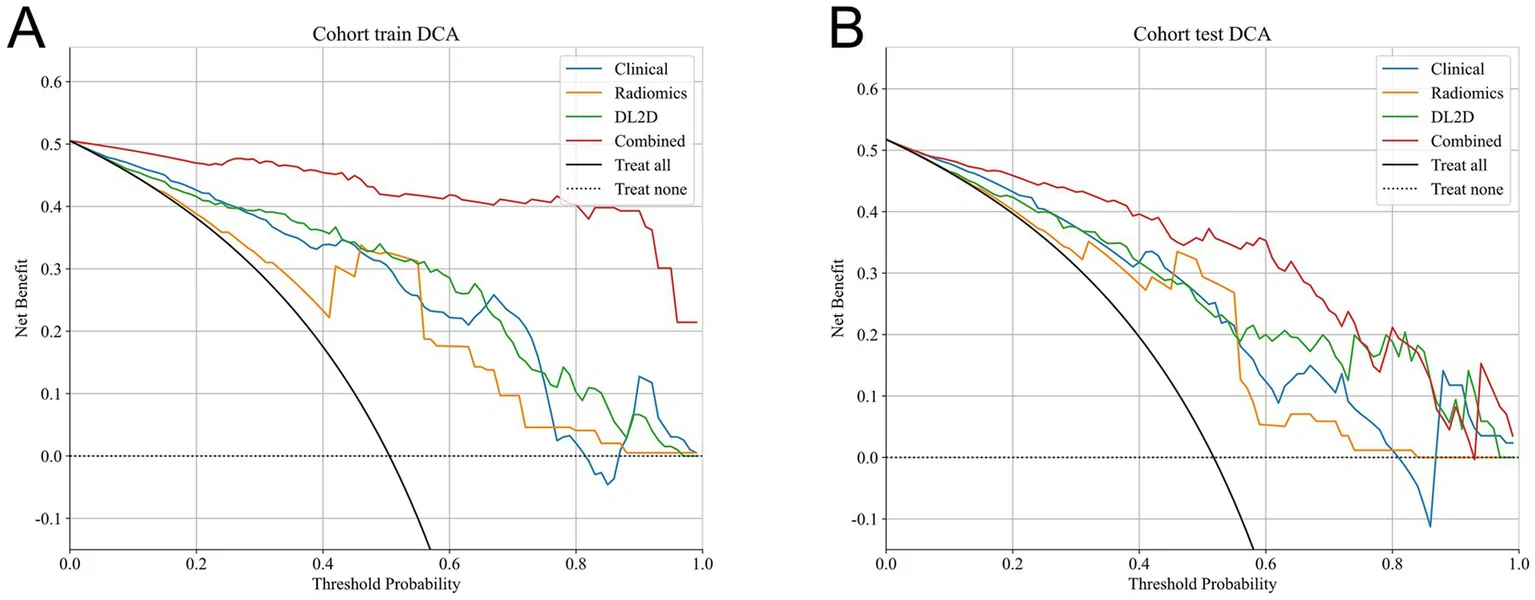
Clinical value assessment of the models. **(A)** DCA curve of the training cohort; **(B)** DCA curve of the testing cohort. DCA, decision curve analysis.

**Figure 4:**
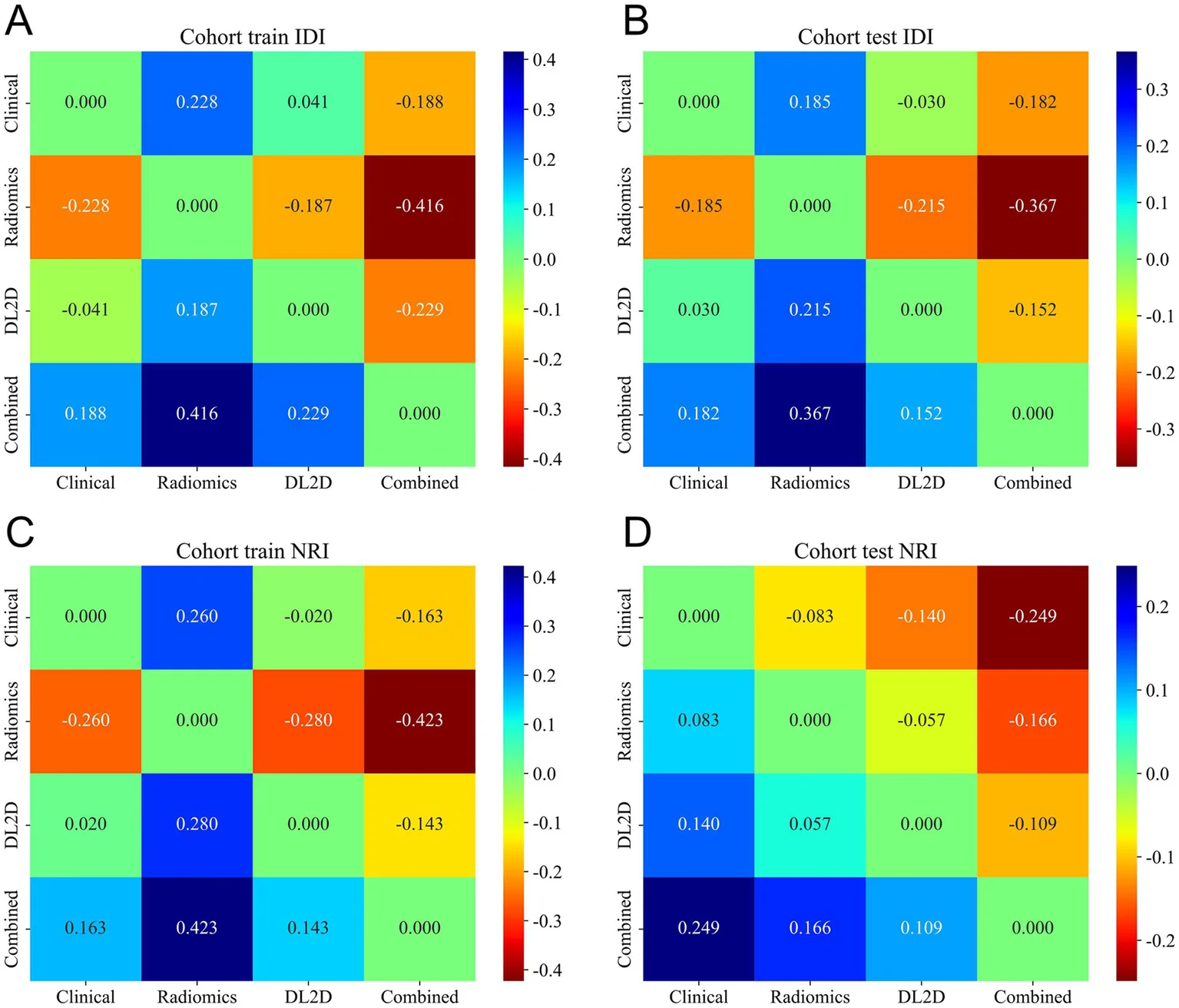
IDI and NRI of different models. **(A)** IDI in training cohort; **(B)** IDI in testing cohort; **(C)** NRI in training cohort; **(D)** NRI in testing cohort. IDI, integrated discrimination improvement; NRI, net reclassification improvement.

Agreement between predicted probabilities and observed outcomes was evaluated via the Hosmer-Lemeshow goodness-of-fit test and calibration plots (Figures 2A,B). The combined model exhibited excellent calibration in both the training and test sets, with Hosmer-Lemeshow test *p* values of 0.092 and 0.280, respectively, indicating satisfactory agreement between predicted and observed outcomes. To determine the statistical significance of differences in diagnostic performance between models, the DeLong test was employed. The combined model consistently outperformed most single-modality models and showed statistically significant improvements over both the radiomics model and the clinical model (*p* < 0.05), supporting the robustness of the multimodal approach. Decision curve analysis (DCA) for the training and test sets is presented in Figures 3A,B. Across a broad range of threshold probabilities, the combined model provided greater net clinical benefit than the other models, further supporting its potential clinical utility. Pairwise comparisons based on the integrated discrimination improvement (IDI) and net reclassification improvement (NRI) further confirmed that, in both the training and test sets, the combined model improved predictive performance relative to the clinical, radiomics, and DL2D models (Figures 4A–D).

## 4 Discussion

TPE and MPE are two of the most common types of exudative PE in clinical practice. Their early and accurate differentiation remains a core clinical challenge in respiratory medicine, infectious diseases, and radiology (19). On conventional chest CT, the two conditions often exhibit highly overlapping morphological features, making stable differentiation difficult based on subjective visual assessment alone. Furthermore, conventional diagnostic approaches, including pleural fluid cytology, pleural biopsy, tumor marker detection, and biochemical analysis, are limited either by their invasive nature and poor patient compliance or by insufficient sensitivity and high false-negative rates. The risks of misdiagnosis and missed diagnosis are significantly elevated, particularly in patients with occult lesions or atypical presentations (20, 21). More critically, the treatment pathways and prognostic trajectories for TPE and MPE differ substantially; diagnostic delay or errors may result in missed therapeutic opportunities, disease progression, and adverse clinical outcomes. Against this background, the development of non-invasive, objective, and reproducible AI-based diagnostic tools has become an important direction for overcoming the limitations of conventional diagnostic strategies. Previous studies have explored the value of radiomics or deep learning models in the differential diagnosis of pleural effusion and have reported encouraging results. For example, Xing et al. included 81 patients, extracted 944 radiomics features from each case, selected 14 optimal features, and built an SVM-based prediction model that achieved AUCs of 0.96 and 0.86 in the training and test sets, respectively (22). Han et al. combined radiomics and clinical features, selecting 19 radiomics features and two clinical variables to construct a combined model with AUCs of 0.968 and 0.873 in the training and test sets, respectively, significantly outperforming the clinical and radiomics models alone (23). Wei et al. further integrated radiomics and CT semantic features, selecting four CT features and 13 radiomic parameters to build a combined model that achieved AUCs of 0.926 and 0.916 in the training and test sets, respectively (24). Despite these promising findings, previous studies have several limitations. First, most relied on manual ROI segmentation, which is time-consuming and labor-intensive and may introduce non-negligible subjective bias because of interobserver variability, thereby affecting model stability and reproducibility. Second, most previously reported models were based primarily on clinical variables and radiomics features, with relatively few studies integrating deep learning into the fusion process. More importantly, many prior studies did not explicitly incorporate precise lesion segmentation as an integral component of the diagnostic workflow and therefore did not establish a true end-to-end “segmentation-diagnosis” framework. In the present study, we developed an integrated framework combining U-Net-based automatic segmentation with deep learning classification and multimodal fusion. The resulting combined model achieved an AUC of 0.934 in the test set, with a sensitivity of 0.875 and a specificity of 0.900, outperforming the standalone clinical, radiomics, and DL2D models. The U-Net module also showed stable segmentation performance, with Dice coefficients above 0.862, indicating that it could localize lesion regions with good consistency. This automated segmentation approach may reduce the workload associated with manual delineation, improve reproducibility, and facilitate future clinical translation by enabling a more automated diagnostic pipeline.

Single-modality models often have inherent limitations. Clinical models are based on structured variables selected by humans and are generally interpretable, but they are unable to capture subtle image features. Radiomics models quantify lesion texture, morphology, and intensity distribution through handcrafted features, but their representation capacity is constrained by predefined feature engineering. By contrast, DL2D models automatically learn high-level image representations in an end-to-end manner, although their performance may depend more strongly on sample size and data diversity. By fusing these three modalities, this study sought to leverage their complementary strengths. Clinical variables provide relevant baseline information, radiomics features contribute quantitative descriptions of lesion heterogeneity, and DL2D features add higher-level image representations. The superior performance of the combined model is likely related to the underlying biologic and pathologic differences between TPE and MPE. TPE is an infectious process in which the pleura typically shows granulomatous inflammation, fibrinous exudation, and fibrous adhesion in response to MTB infection. On imaging, these processes may appear as diffuse pleural thickening, relatively homogeneous involvement, and more regular effusion characteristics (2, 25). In contrast, MPE results from pleural dissemination of tumor cells or primary malignant pleural tumors. The pleura may show nodular or mass-like proliferation and irregular enhancement, with imaging findings such as pleural nodularity, lobulated thickening, and heterogeneous pleural abnormalities (26, 27). These pathological differences likely translate into multidimensional differences in texture, morphology, edge features, and intensity distribution on medical images. The clinical model reflects differences in epidemiologic background and systemic inflammatory responses, the radiomics model captures quantitative imaging manifestations of lesion heterogeneity, and the DL2D model further extracts higher-order semantic information that may not be visually apparent. By integrating these sources of information, the combined model achieves a more comprehensive characterization of the disease and thereby improves discriminatory performance. It is worth noting that in the feature importance analysis, the radiomics features accounted for the highest overall weight in the comprehensive model. This is consistent with the result that the radiomics model independently performed better (AUC = 0.841) than the DL2D model (AUC = 0.776) and the clinical model (AUC = 0.767). This finding suggests that the texture features of CT images (such as GLCM entropy and GLSZM large area low gray-level emphasis) may more directly capture the microscopic differences between TPE and MPE at the pathological level, such as the degree of necrosis, the distribution of fibrosis, and the spatial heterogeneity of cell density. At the same time, clinical variables (especially age and CD4 + T cell count), although they have limited predictive efficacy when used independently, still contributed approximately 20% of the importance weight in the multimodal fusion, indicating that they provide information on the host immune status and epidemiological background that is difficult for imaging to capture. This complementary fusion mechanism further confirms the scientific rationale of the multimodal strategy.

Several limitations should be acknowledged. First, this was a retrospective single-center study, and although the sample size was sufficient for initial model development, broader validation is still needed. Future multicenter prospective studies are required to further assess robustness and generalizability. Second, U-Net training still depended on manually annotated lesion regions. Although annotation was performed by experienced radiologists, some degree of subjectivity remains unavoidable. Weakly supervised or self-supervised segmentation methods may help reduce dependence on finely annotated data in future work. Third, the combined model integrated imaging and clinical data but did not incorporate molecular biomarkers such as gene expression profiles or circulating tumor DNA. With the continued development of multi-omics approaches, future studies may explore radiogenomics or other multimodal strategies to better clarify the relationship between molecular mechanisms and imaging phenotypes.

## Data Availability

The original contributions presented in the study are included in the article/Supplementary material, further inquiries can be directed to the corresponding authors.
