# Supplementary material for: Differential diagnosis model for tuberculous and malignant pleural effusion combining U-Net automatic segmentation and deep learning
Source: Front Med (Lausanne). 2026 Sep 1;13:1855938. doi: 10.3389/fmed.2026.1855938 (PMC13574948; doi:10.3389/fmed.2026.1855938)
Supplement: Supplementary file 1 [file Supplementary_file_1.docx]

Supplementary Material

**Supplementary Table 1.** Baseline characteristic.

| Feature Name | ALL | Train | Test | P |
| --- | --- | --- | --- | --- |
| age | 62.23±15.94 | 61.59±16.08 | 63.72±15.60 | 0.324 |
| height | 162.14±7.73 | 162.73±7.78 | 160.79±7.50 | 0.032 |
| weight | 54.09±10.42 | 54.48±10.45 | 53.20±10.36 | 0.176 |
| BMI | 20.51±3.32 | 20.52±3.45 | 20.48±3.02 | 0.851 |
| CD3 | 804.59±403.88 | 786.86±382.47 | 845.49±449.13 | 0.192 |
| CD4 | 425.73±255.50 | 418.76±252.04 | 441.79±264.13 | 0.238 |
| CD8 | 332.82±245.10 | 322.29±211.54 | 357.11±309.05 | 0.732 |
| CD4/CD8 | 1.72±1.34 | 1.71±1.45 | 1.73±1.04 | 0.432 |
| WBC | 9.08±9.02 | 8.58±7.48 | 10.23±11.81 | 0.158 |
| RBC | 3.97±2.09 | 4.01±2.43 | 3.87±0.90 | 0.867 |
| hemoglobin | 109.79±70.02 | 105.39±25.14 | 119.95±121.36 | 0.65 |
| platelet | 289.53±127.94 | 282.24±136.09 | 306.33±105.66 | 0.021 |
| neutrophil | 6.37±4.35 | 6.14±4.21 | 6.91±4.65 | 0.197 |
| lymphocyte | 1.22±0.69 | 1.19±0.69 | 1.28±0.70 | 0.344 |
| monocyte | 0.59±0.33 | 0.58±0.36 | 0.62±0.26 | 0.216 |
| eosinophils | 0.24±0.47 | 0.24±0.49 | 0.26±0.43 | 0.885 |
| basophilia | 0.04±0.06 | 0.04±0.07 | 0.04±0.03 | 0.763 |
| CRP | 46.35±50.43 | 44.42±47.30 | 50.79±57.05 | 0.608 |
| ESR | 63.56±39.53 | 61.77±38.17 | 67.71±42.46 | 0.346 |
| K | 3.83±0.65 | 3.84±0.60 | 3.81±0.75 | 0.233 |
| Na | 137.42±7.48 | 137.04±8.36 | 138.29±4.79 | 0.347 |
| Ca | 2.50±6.02 | 2.66±7.21 | 2.11±0.17 | 0.179 |
| CREA | 104.82±159.58 | 110.65±168.74 | 91.37±136.11 | 0.098 |
| GLU | 6.29±2.36 | 6.46±2.38 | 5.89±2.28 | 0.022 |
| TG | 1.31±0.70 | 1.31±0.72 | 1.33±0.66 | 0.483 |
| AST | 25.58±36.67 | 23.09±27.79 | 31.33±51.40 | 0.292 |
| ALB | 33.51±6.61 | 33.81±6.56 | 32.82±6.71 | 0.25 |
| gender |  |  |  | 0.017 |
| Male | 192(68.33) | 143(72.96) | 49(57.65) |  |
| Female | 89(31.67) | 53(27.04) | 36(42.35) |  |
| hypertension |  |  |  | 0.456 |
| No | 221(78.65) | 157(80.10) | 64(75.29) |  |
| Yes | 60(21.35) | 39(19.90) | 21(24.71) |  |
| diabetes |  |  |  | 1.0 |
| No | 254(90.39) | 177(90.31) | 77(90.59) |  |
| Yes | 27(9.61) | 19(9.69) | 8(9.41) |  |
| COPD |  |  |  | 0.874 |
| No | 267(95.02) | 187(95.41) | 80(94.12) |  |
| Yes | 14(4.98) | 9(4.59) | 5(5.88) |  |
| heart disease |  |  |  | 0.86 |
| No | 178(63.35) | 123(62.76) | 55(64.71) |  |
| Yes | 103(36.65) | 73(37.24) | 30(35.29) |  |
| hepatitis |  |  |  | 0.577 |
| No | 259(92.17) | 179(91.33) | 80(94.12) |  |
| Yes | 22(7.83) | 17(8.67) | 5(5.88) |  |
| anemia |  |  |  | 0.934 |
| No | 209(74.38) | 145(73.98) | 64(75.29) |  |
| Yes | 72(25.62) | 51(26.02) | 21(24.71) |  |
| coagulation disorders |  |  |  | 0.347 |
| No | 256(91.10) | 176(89.80) | 80(94.12) |  |
| Yes | 25(8.90) | 20(10.20) | 5(5.88) |  |
| cerebral infarction |  |  |  | 0.522 |
| No | 242(86.12) | 171(87.24) | 71(83.53) |  |
| Yes | 39(13.88) | 25(12.76) | 14(16.47) |  |
| HIV |  |  |  | 1.0 |
| No | 248(88.26) | 173(88.27) | 75(88.24) |  |
| Yes | 33(11.74) | 23(11.73) | 10(11.76) |  |
| smoke |  |  |  | 1.0 |
| No | 218(77.58) | 152(77.55) | 66(77.65) |  |
| Yes | 63(22.42) | 44(22.45) | 19(22.35) |  |
| drink |  |  |  | 0.186 |
| No | 230(81.85) | 156(79.59) | 74(87.06) |  |
| Yes | 51(18.15) | 40(20.41) | 11(12.94) |  |
| fever |  |  |  | 0.603 |
| No | 241(85.77) | 170(86.73) | 71(83.53) |  |
| Yes | 40(14.23) | 26(13.27) | 14(16.47) |  |
| chest distress |  |  |  | 0.555 |
| No | 210(74.73) | 144(73.47) | 66(77.65) |  |
| Yes | 71(25.27) | 52(26.53) | 19(22.35) |  |
| cough |  |  |  | 0.159 |
| No | 97(34.52) | 62(31.63) | 35(41.18) |  |
| Yes | 184(65.48) | 134(68.37) | 50(58.82) |  |
| expectoration |  |  |  | 0.735 |
| No | 123(43.77) | 84(42.86) | 39(45.88) |  |
| Yes | 158(56.23) | 112(57.14) | 46(54.12) |  |
| chest pain |  |  |  | 0.626 |
| No | 228(81.14) | 161(82.14) | 67(78.82) |  |
| Yes | 53(18.86) | 35(17.86) | 18(21.18) |  |
| breathing difficulties |  |  |  | 0.057 |
| No | 247(87.90) | 167(85.20) | 80(94.12) |  |
| Yes | 34(12.10) | 29(14.80) | 5(5.88) |  |
| moist rales |  |  |  | 0.88 |
| No | 182(64.77) | 128(65.31) | 54(63.53) |  |
| Yes | 99(35.23) | 68(34.69) | 31(36.47) |  |

**Supplementary Table 2.** Dice of each model in train and test cohort

| Dice | mIOU | SA | OS | US | Cohort |
| --- | --- | --- | --- | --- | --- |
| 0.873 | 0.823 | 0.877 | 0.071 | 0.081 | train |
| 0.862 | 0.805 | 0.876 | 0.080 | 0.086 | test |

**Supplementary Table 3.** Univariable and multivariable analysis of clinical features.

| Feature Name | OR | OR lower 95%CI | OR upper 95%CI | P | OR | OR lower 95%CI | OR upper 95%CI | P |
| --- | --- | --- | --- | --- | --- | --- | --- | --- |
| breathing difficulties | 0.624 | 0.533 | 0.731 | 0.000 | 0.755 | 0.658 | 0.866 | 0.001 |
| HIV | 0.654 | 0.548 | 0.781 | 0.000 | 0.648 | 0.551 | 0.763 | 0.0 |
| cough | 0.659 | 0.586 | 0.741 | 0.000 | 1.022 | 0.87 | 1.2 | 0.825 |
| COPD | 0.662 | 0.501 | 0.875 | 0.015 | 0.735 | 0.59 | 0.917 | 0.023 |
| chest distress | 0.671 | 0.592 | 0.760 | 0.000 | 0.876 | 0.784 | 0.979 | 0.05 |
| expectoration | 0.693 | 0.620 | 0.776 | 0.000 | 0.713 | 0.614 | 0.827 | 0.0 |
| chest pain | 0.793 | 0.680 | 0.923 | 0.013 | 0.854 | 0.754 | 0.967 | 0.036 |
| smoke | 0.833 | 0.724 | 0.959 | 0.033 | 1.013 | 0.908 | 1.131 | 0.845 |
| hepatitis | 0.847 | 0.687 | 1.044 | 0.191 |  |  |  |  |
| drink | 0.876 | 0.757 | 1.014 | 0.138 |  |  |  |  |
| hypertension | 0.888 | 0.766 | 1.030 | 0.187 |  |  |  |  |
| gender | 0.907 | 0.794 | 1.037 | 0.227 |  |  |  |  |
| coagulation disorders | 0.940 | 0.773 | 1.143 | 0.605 |  |  |  |  |
| lymphocyte | 0.954 | 0.875 | 1.040 | 0.366 |  |  |  |  |
| monocyte | 0.970 | 0.823 | 1.143 | 0.762 |  |  |  |  |
| GLU | 0.974 | 0.950 | 0.998 | 0.081 |  |  |  |  |
| RBC | 0.975 | 0.952 | 0.999 | 0.090 |  |  |  |  |
| BMI | 0.976 | 0.960 | 0.993 | 0.022 | 0.994 | 0.98 | 1.009 | 0.503 |
| hemoglobin | 0.994 | 0.992 | 0.996 | 0.000 | 0.997 | 0.995 | 0.999 | 0.034 |
| WBC | 0.995 | 0.987 | 1.003 | 0.341 |  |  |  |  |
| age | 0.996 | 0.992 | 1.000 | 0.064 |  |  |  |  |
| ALB | 0.996 | 0.987 | 1.005 | 0.496 |  |  |  |  |
| weight | 0.997 | 0.991 | 1.002 | 0.314 |  |  |  |  |
| Na | 0.997 | 0.990 | 1.004 | 0.433 |  |  |  |  |
| neutrophil | 0.998 | 0.984 | 1.012 | 0.803 |  |  |  |  |
| platelet | 1.000 | 0.999 | 1.000 | 0.224 |  |  |  |  |
| CD4/CD8 | 1.000 | 0.960 | 1.042 | 0.996 |  |  |  |  |
| AST | 1.000 | 0.998 | 1.002 | 0.811 |  |  |  |  |
| CD4 | 1.000 | 1.000 | 1.000 | 0.395 |  |  |  |  |
| CD3 | 1.000 | 1.000 | 1.000 | 0.170 |  |  |  |  |
| CD8 | 1.000 | 0.999 | 1.000 | 0.050 | 1.0 | 1.0 | 1.0 | 0.937 |
| CRP | 1.001 | 1.000 | 1.002 | 0.284 |  |  |  |  |
| CREA | 1.001 | 1.000 | 1.001 | 0.002 | 1.001 | 1.0 | 1.001 | 0.011 |
| ESR | 1.001 | 0.999 | 1.002 | 0.513 |  |  |  |  |
| Ca | 1.005 | 0.997 | 1.013 | 0.325 |  |  |  |  |
| TG | 1.007 | 0.927 | 1.093 | 0.897 |  |  |  |  |
| height | 1.009 | 1.001 | 1.016 | 0.061 |  |  |  |  |
| diabetes | 1.024 | 0.838 | 1.251 | 0.847 |  |  |  |  |
| K | 1.029 | 0.931 | 1.135 | 0.638 |  |  |  |  |
| moist rales | 1.038 | 0.917 | 1.176 | 0.622 |  |  |  |  |
| cerebral infarction | 1.065 | 0.891 | 1.273 | 0.559 |  |  |  |  |
| eosinophils | 1.069 | 0.948 | 1.206 | 0.359 |  |  |  |  |
| fever | 1.187 | 0.998 | 1.412 | 0.104 |  |  |  |  |
| anemia | 1.244 | 1.090 | 1.420 | 0.007 | 1.036 | 0.919 | 1.166 | 0.627 |
| heart disease | 1.275 | 1.132 | 1.436 | 0.001 | 1.192 | 1.08 | 1.317 | 0.004 |
| basophilia | 1.577 | 0.638 | 3.896 | 0.407 |  |  |  |  |

**Supplementary Figure 1.** Dice of training process.

**Supplementary Figure 2.** Workflow for the feature selection process in radiomics. (A. Coefficients of 10 folds cross validation; B. MSE of 10 folds cross validation; C. The histogram of the Rad-score based on the selected features)
